# Medical image segmentation with UNet-based multi-scale context fusion

**DOI:** 10.1038/s41598-024-66585-x

**Published:** 2024-10-28

**Authors:** Yongqi Yuan, Yong Cheng

**Affiliations:** School of Information Technology, Jiangsu Open University, Nanjing, 210000 Jiangsu China

**Keywords:** Computational biology and bioinformatics, Oncology, Computer science

## Abstract

Histopathological examination holds a crucial role in cancer grading and serves as a significant reference for devising individualized patient treatment plans in clinical practice. Nevertheless, the distinctive features of numerous histopathological image targets frequently contribute to suboptimal segmentation performance. In this paper, we propose a UNet-based multi-scale context fusion algorithm for medical image segmentation, which extracts rich contextual information by extracting semantic information at different encoding stages and assigns different weights to the semantic information at different scales through TBSFF module to improve the learning ability of the network for features. Through multi-scale context fusion and feature selection networks, richer semantic features and detailed information are extracted. The target can be more accurately segmented without significantly increasing the extra overhead. The results demonstrate that our algorithm achieves superior Dice and IoU scores with a relatively small parameter count. Specifically, on the GlaS dataset, the Dice score is 90.56, and IoU is 83.47. For the MoNuSeg dataset, the Dice score is 79.07, and IoU is 65.98.

## Introduction

Pathological examinations are typically conducted by pathologists who scrutinize stained specimens through a microscope, a process demanding extensive medical expertise and professional training^1^. Throughout this examination, pathologists must concentrate on pivotal areas of significance within tissue samples, alongside randomly selected sub-areas. Subsequently, they render judgments to ascertain whether the sample is normal or pathological. However, histopathological images frequently encompass a multitude of targets, presenting a challenge for traditional image segmentation algorithms that tend to exhibit subpar performance in handling such images. With the revival and prosperity of deep learning technology in the field of computer vision, the application of methods such as image classification^2,3^, image segmentation^4,5^, image positioning and application based on deep learning in natural scenes and medical imaging has also increased dramatically. The widespread adoption of deep learning in medical imaging owes to its proficiency in image interpretation and classification, offering innovative approaches to medical image segmentation. Medical image segmentation stands as a pivotal technology for the analysis and comprehension of medical images, delivering crucial auxiliary information for clinicians in diagnosis, treatment, and contributing significantly to disease monitoring and predicting treatment outcomes. In tandem with the rapid advancements in medical imaging technology, histopathology has experienced substantial progress, marked by the increasing prevalence of deep learning applications in medical image segmentation^6^. This trend provides a promising solution for the intricate task of segmenting numerous targets in histopathology.

Among various network models, UNet stands out as the most widely used encoder–decoder model for medical image segmentation^7^. The encoder captures low and high semantic features, while the decoder reconstructs the final result by leveraging these captured features. To recover spatial information lost during pooling, skip connections are employed. However, current state-of-the-art semantic segmentation methods often rely on simple merging of low-semantic features from different levels, lacking effective extraction of the most informative parts from various semantic levels.

UNet++, a well-known variant of the UNet network, addresses this limitation by narrowing the semantic gap between the encoder and decoder. It achieves this by introducing dense skip connections that incorporate more low-semantic features from the encoder into the decoder, leading to improved segmentation performance^8^. This method increases the number of skip connections and maximizes the fusion of low-semantic features with other semantic features of the same size.

An alternative approach involves the use of Transformer-based methods, with UCTransNet being a notable representative^9^. Instead of simply merging low-semantic information from the encoder with high-semantic information from the decoder via skip connections, this approach first passes the low-semantic information through two Transformer-based modules, CCT (Channel Cross fusion with Transformer) and CCA (Channel-wise Cross-Attention). These modules perform fusion of different levels of semantic information concurrently and exhibit similarities to channel attention mechanisms.

While previous methods have demonstrated notable improvements in image segmentation performance, they often come with a substantial increase in model parameters and struggle to effectively extract crucial information from low semantic features. We contend that the UNet encoder alone is adept at feature extraction. However, the primary limitation of UNet lies in its skip connections. Thus, we propose the TBSFF-UNet (Three-Branch Feature Fusion UNet) model, which introduces novel skip connections to integrate diverse semantic levels. This facilitates the aggregation of information, enabling the capture of richer semantic details even at lower feature layers, subsequently forwarded to the decoder module for deeper analysis and processing. Additionally, we introduce a three-branch feature fusion module (TBSFF) for selecting semantic information from different levels. Employing a dynamic selection mechanism, the TBSFF module enhances segmentation performance by extracting pertinent information from the three input branches with varying degrees of attention. Our contributions can be summarized as follows:

1. The skip connections in the network are redesigned to achieve efficient use of features, thereby ensuring that the features of the input image can be effectively transferred to the decoder module for further processing and analysis.
2. TBSFF (three-branch feature fusion network): The TBSFF module serves as a dynamic selection mechanism, enhancing segmentation effectiveness by extracting pertinent information from the three input branches with varying degrees of attention. This proposed TBSFF module facilitates the fusion of multi-scale features, which are subsequently transmitted to the encoder module. Such an approach significantly augments the model’s capability to capture information across various scales.
3. Performance evaluation: Through experiments conducted on the MoNuSeg and GlaS public medical datasets, we assess the performance of TBSFF-UNet. Particularly, we select a subset of challenging images and observe that TBSFF-UNet exhibits a significant improvement over UNet when applied to these specific cases.

## Related works

Long et al.^10^ were the pioneers in introducing a fully convolutional network. This groundbreaking approach replaced the fully connected layer of traditional convolutional neural networks (CNNs) with deconvolution operations, enabling pixel-level predictions for images. The advent of fully convolutional networks in the realm of image semantic segmentation has played a pivotal role in advancing the field of image segmentation and has yielded remarkable outcomes, particularly in the domain of medical image segmentation.

Ronnebergerder et al.^7^ proposed the widely recognized UNet architecture, which remains a cornerstone in the field of medical image segmentation. The UNet model comprises two key components: an encoder and a decoder. The encoder consists of five stages, where basic convolution operations are applied, followed by ReLU activation function. Subsequently, a 2 $\times$ 2 pooling operation is employed to downsample the image for the first four stages. In the decoder, the encoded features are utilized, and an upsampling operation is performed to gradually recover the spatial and detailed information of the image. By extending the fully convolutional network, the UNet model enhances the network depth, progressively reduces the size of the feature map through additional convolutional layers, and effectively integrates contextual information using skip connections. This novel network structure has garnered significant attention, leading to the proposal of various modified architectures based on UNet^11–15^.

Drawing inspiration from the UNet network, Zhou et al. introduced the UNet++ structure, which represents a more potent architecture for medical image segmentation. The fundamental essence of this architecture remains the classic encoder–decoder network. However, unlike the original UNet, the UNet++ network incorporates a series of nested dense skip connections. These redesigned skip connections exploit feature maps from the encoder more effectively, thereby reducing the semantic gap between the encoder and decoder. Consequently, the model captures fine-grained image details, leading to improved segmentation outcomes. Building upon the UNet++ architecture, Zhou et al.^16^ further enhanced the skip connections to aggregate features of different semantic scales within the decoder sub-network, resulting in a highly flexible feature fusion scheme.

In addition to UNet and UNet++, other innovative architectures have been proposed in the field of medical image segmentation. For instance, Alom et al.^17^ introduced the R2U-Net network, which combines the advantages of U-Net, residual networks, and recurrent CNNs. This architecture leverages residual connections to increase network depth and recurrent CNNs to extract feature information more effectively from images. The R2U-Net demonstrates superior performance over UNet in various tasks such as blood vessel segmentation, skin cancer segmentation, and lung lesion segmentation in retinal images. Similarly, Oktay et al.^18^ presented an attention-based model named attention U-Net for medical image segmentation. This model automatically learns to prioritize objects of different shapes and sizes, assigning different weights to relevant and irrelevant regions. By highlighting the useful features of relevant regions and suppressing the invalid features of unrelated regions, the attention U-Net improves model accuracy without significantly increasing computational costs.

Although these architectures exhibit certain differences, they collectively demonstrate the value of reducing the semantic discrepancy between the encoder and decoder through skip connections, leading to improved performance of the UNet model. However, it is important to note that introducing excessive skip connections may result in increased network complexity and a larger model size. With the integration of Transformer architecture into computer vision, the introduction of Vision Transformer (ViT)^19^ has yielded remarkable results on various image recognition benchmarks, such as ImageNet, CIFAR-100, and VTAB. By applying the Transformer’s self-attention mechanism to image classification, ViT has achieved superior performance. Inspired by ViT’s success in computer vision, several Transformer-based models for image segmentation have emerged^20–24^. TransUNet^25^ was the first Transformer-based medical image segmentation model, combining the strengths of Transformer and UNet. The model utilizes Transformer to encode tokenized image patches from CNN feature maps, extracting global contextual information. Additionally, the encoder performs an upsampling operation on the feature map after the Transformer, which is then combined with the CNN-encoded feature map to gradually restore the spatial and detailed information of the image. Valanarasu et al. introduced Med Transformer^26^, a Transformer-based model that incorporates a gated axial-attention mechanism in the self-attention module. The architecture includes an additional control mechanism, facilitating efficient computation of context information and enabling long-range interactions in feature map encoding. The authors also proposed a local-global training strategy, employing two branches: a local branch for detailed information at the original image resolution and a global branch for high-level information. This approach achieves promising results across three different datasets.

Cao et al. presented Swin-Unet^27^, a pure Transformer-based model similar to UNet. Swin-Unet utilizes a hierarchical Transformer with a shifted window as a decoder to extract contextual features, replacing the convolutional layers in the UNet network. The model also incorporates a Transformer-based decoder to perform the deconvolution operation and recover spatial information in the feature map. By designing skip connections, the model achieves effective contextual information interaction, demonstrating favorable results on multi-organ and cardiac segmentation datasets. Wang et al. proposed the UCTransNet model^19^, comprising a multi-scale cross-channel fusion (CCT) and an effectively connected cross-channel attention interaction sub-module (CCA) for guiding fusion. The model incorporates attention mechanisms and leverages the CCT and CCA modules as replacements for the skip connections in the UNet network. UCTransNet has achieved promising results on the GlaS, MoNuSeg, and Synapse datasets. Wang et al.^28^ introduced the Swin Deformable Attention Hybrid UNet (SDAH-UNet) model, incorporating the Swin Deformable model. This model not only enhances segmentation performance but also offers a more direct interpretation for convolutional and multi-head self-attention mechanisms. Liao et al.^29^ proposed the Transformer-based Annotation-Bias-Aware (TAB) model, which addresses annotation bias by considering bias in the labeling process. The model models the relationship between annotators’ preferences and random errors to mitigate annotator-related biases. To overcome the “token-flatten” challenge associated with the direct utilization of the Transformer, He et al.^30^ introduced U-Nermer. This method involves segmenting the input image into local patches, with the global contextual information among these patches being learned through the self-attention mechanism employed by Transformer and UNet.

The proposal of these networks has extensively promoted the development of medical image segmentation technology. Unfortunately, these networks generally have the problems of large models, large model parameters, and large FlOPs, and it is difficult to achieve a good relationship between the effect and the model complexity balance.

## Proposed network architecture: TBSFF-UNet

### Overall architecture

U-Net stands as a cornerstone in medical image segmentation, garnering significant attention from researchers. In the realm of histological image segmentation, numerous scholars have endeavored to enhance U-Net at various levels. Yet, to our knowledge, existing improvements to U-Net predominantly concentrate on bolstering its encoder. For instance, TransUNet^25^ adopts a simplistic approach by integrating Transformer into U-Net’s encoder module, while Swin-UNet^27^ pioneers the introduction of Swin Transformer to replace the convolution block in U-Net, forming the first U-shaped architecture purely based on Transformer. However, we maintain that merely integrating Transformer into U-Net to enhance the encoder’s information extraction capability may not yield optimal network performance. Instead, it tends to exacerbate model complexity, taxing computational resources and impeding network training. We posit that U-Net’s encoder suffices for feature extraction. The principal bottleneck in U-Net’s efficacy lies in its skip connections. Therefore, our proposed TBSFF-UNet model seeks to ameliorate network performance by refining skip connections without substantially augmenting model complexity.

**Figure 1:**
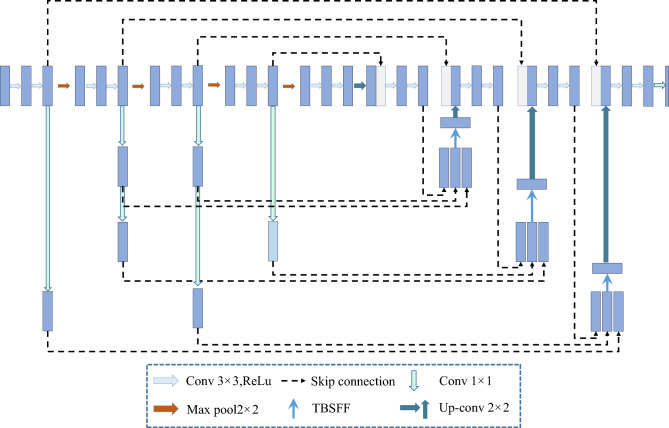
Illustration of the proposed TBSFF-UNet.

In this work, we propose a novel network architecture, termed TBSFF-UNet, illustrated in Fig. 1. The network comprises an encoder, decoder, and feature fusion module. The encoder learns from input images, extracts features through encoding at different stages, and transmits them to both the decoder module and the TBSFF module through skip connections. TBSFF-UNet redefines skip connections in the network to efficiently utilize features.

By designing a new network structure, TBSFF-UNet optimizes the feature transfer between the encoder and decoder. The TBSFF-UNet module then performs multi-branch feature fusion on different features, extracting more critical features. During this process, TBSFF employs a kernel selection mechanism to model features from different levels passed between the encoder and decoder. It generates different weights to adjust and fuse features from different levels, producing new and more effective features for improved segmentation results. Importantly, TBSFF-UNet achieves enhanced segmentation performance without significantly increasing model parameters and computational complexity, thereby ensuring model efficiency alongside improved segmentation outcomes.

### Redesigned skip connection path

In the U-Net architecture, the encoder’s features are passed to the decoder through skip connections, effectively reducing the semantic gap between the encoder and decoder. In U-Net++, the encoder’s feature maps undergo dense convolutional blocks, and a substantial number of skip connections are employed to transmit high-resolution features from the encoder to the decoder, enhancing the proximity of semantic features between the encoder and decoder. However, this approach significantly increases the model’s complexity. In the context of histological image segmentation tasks, we observed that more skip connections do not necessarily lead to better results; instead, it is crucial to design appropriate skip connections.

In this work, we present the TBSFF-UNet network, a modification of the U-Net architecture that redefines skip connections, as illustrated in Fig. 1. Compared to U-Net++, TBSFF-UNet reduces the number of skip connections considerably. It achieves this by transmitting the feature maps from both the encoder and the partially decoded feature maps from the decoder to the TBSFF module. This process aids in extracting effective features, thereby enhancing segmentation precision. Figure 1 also demonstrates that TBSFF-UNet’s skip connections, in comparison to U-Net++, only introduce a limited number of additional connections.

Furthermore, the skip connections designed in this study not only transmit features from the corresponding decoder stages in the encoder to the decoder but also convey features from the previous and subsequent encoder stages to the TBSFF module. This approach enables the transfer of more low-level, high-resolution features to TBSFF, helping alleviate spatial information loss caused by the encoding stage.

### Redesigned selective kernel networks

**Figure 2:**
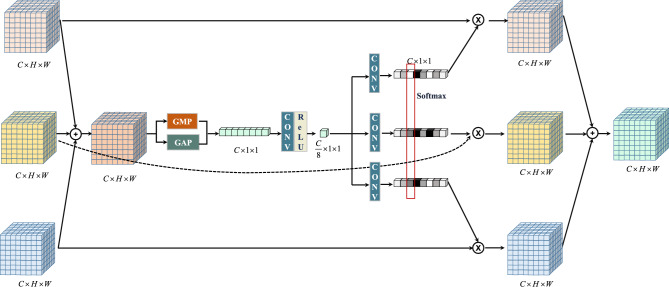
Illustration of the proposed TBSFF.

The TBSFF-UNet model proposed in this paper does not simply concatenate the features passed through skip connections from the encoder and the features from the decoder. Instead, it employs a selectively designed three-branch fusion module for merging the input three feature maps, known as TBSFF, as depicted in Fig. 2. This module represents a dynamic selection mechanism where attention to different branches varies. The selective convolution is achieved through the Fuse and Select operations, implementing a mechanism for dynamic feature fusion.

The basic idea of the TBSFF module is to selectively control the flow of semantic information from 3 branches at different scales to the next neuron^31,32^. To achieve this task, the information from the three branches is mixed. (1) FUSE: Combine these features by element summation $\mathrm{{U = SUM(}}{\mathrm{{U}}_1}\mathrm{{,}}{\mathrm{{U}}_2},{U_3})$, perform global max pooling and global average pooling in the dimension of $H \times W \times C$ to calculate channel statistics $s \in {R^{1 \times 1 \times C}}$, then use the ${\textrm{Re}} LU(Conv( \cdot ))$ reduced dimension of the feature map. Finally, the feature map increases the dimension through three parallel channels, and three feature descriptors ${W_1}$, ${W_2}$, ${W_3}$ are generated, each of which is $1 \times 1 \times C$. (2) Select: Apply the Softmax function to ${W_1}$, ${W_2}$, ${W_3}$, generate ${T_1}$, ${T_2}$, ${T_3}$, and then act on the three inputs ${U_1}$, ${U_2}$, ${U_3}$. The process is also equivalent to applying an attention mechanism to ${U_1}$, ${U_2}$, ${U_3}$, and using them to selectively calibrate ${U_1}$, ${U_2}$, ${U_3}$, defined as $U = {U_1} \times {T_1} + \mathrm{{ }}{U_2} \times {T_2} + {U_3} \times {T_3}$.

## Experiments

### Datasets

We assess the performance of our proposed model using the gland segmentation benchmark datasets: GlaS^33^ and MoNuSeg^34^. The GlaS dataset comprises 165 images from T3 and T4 stages of 16 H &E stained tissue sections of colorectal adenocarcinoma. It consists of the Training Part, Test Part A, and Test Part B. For this study, 85 images from the training part are utilized for training, while 80 images from test parts A and B are employed for testing. The MoNuSeg dataset comprises 44 images, with 30 designated for training and 14 for testing. These images capture a variety of nuclear appearances from different patients, disease states, and organs, and the dataset includes over 21,000 meticulously annotated nuclear boundaries.

### Implementation details

The network model was implemented using PyTorch, with hardware consisting of dual Intel Xeon E5-2678 v3 @ 2.50GHz CPUs, 32 GB of RAM, and a single Nvidia RTX 3080 GPU card with 10GB of VRAM. For the Glas and MoNuSeg datasets, the batch size was set to 4, the number of epochs was set to 2000, and early stopping was configured after 200 epochs. The input resolution was set to 224 $\times$ 224, patch size was set to 16, and the Adam optimizer was used with a learning rate of 0.001. Binary cross-entropy and dice loss functions were employed as the training loss functions. To ensure experiment reproducibility, CUDA and Python seeds were set. The evaluation during the experiment used the dice coefficient and Intersection over Union (IOU) as performance metrics. To eliminate the randomness of a single experiment, a fivefold cross-validation was conducted, and the results were averaged, along with calculating the standard deviation.

### Loss function

According to the characteristics of binary cross entropy and Dice similarity coefficient, this paper designs a hybrid loss function, in which the value of $\alpha$ is 0.5 and the $\beta$ value of is 0.5. The formula is as follows:

1 $$\begin{aligned} L = \alpha {L_{Dice}} + \beta {L_{BCE}}. \end{aligned}$$

In the two-class medical image segmentation task, the commonly used loss function is the binary cross entropy loss function

2 $$\begin{aligned} {L_{\mathrm{{BCE}}}} = - \frac{1}{N}\mathop \sum \limits _{i = 1}^N \left( {{g_i} \cdot \ln \left( {{p_i}} \right) + \left( {1 - {g_i}} \right) \cdot \ln \left( {1 - {p_i}} \right) } \right) , \end{aligned}$$

where ${g_i}$ is the real category of pixel I, and ${p_i}$ is the prediction of the corresponding pixel.

The binary cross-entropy loss function can effectively solve the problem of the disappearance of the network gradient. Because the loss function performs the same evaluation for each category, it is easy for images with more categories to change for images with unbalanced categories, and the optimization direction of the network also affects the experimental results. Another loss function used is the Dice loss function.

3 $$\begin{aligned} {L_{Dice}} = 1 - \frac{{2\mathop \sum _{i = 1}^N {g_i}{p_i}}}{{\mathop \sum _{i = 1}^N {g_i} + \mathop \sum \limits _{i = 1}^N {p_i}}}. \end{aligned}$$

The Dice loss function is the most common in medical image segmentation, and it is more suitable for unbalanced sample distribution. However, its training error curve is very confusing, making it difficult to get information about convergence.

### Comparison with advanced methods

To verify the performance of the proposed TBSFF-UNet model, we compare it with other CNN-based methods and Transformer-based methods, including UNet, Attention UNet, UNet++, and UCTransNet, which adopt their original released code. The comparison of our experimental results and model complexity is shown in Table 1, where the best results are shown in bold. As can be seen from Table 1, TBSFF-UNet has 17% higher Param and 11% higher FlOPs than UNet. TBSFF-UNet has 57% lower Param and 75% lower FlOPs compared to UNet++, 55% lower Param and 48% lower FlOPs compared to attUNet, and 20% lower Param and FlOPs compared to UCTransNet Param is reduced by 76%, and FlOPs are reduced by 20%. TBSFF-UNet achieves the best results on both the GlaS and MoNuSeg datasets, which shows that the TBSFF-UNet network designed in this paper has good and reliable performance while maintaining light weight.

**Table 1:** Fivefold cross-validation results of each model on the GlaS and MoNuSeg datasets.

Method	Param (M)	FlOPs (G)	GlaS		MoNuSeg	
			Dice (%)	IoU (%)	Dice (%)	IoU (%)
UNet^7^	13.40	23.74	89.42 ± 0.80	81.77 ± 1.31	77.53 ± 0.45	64.38 ± 0.53
UNet++^16^	36.63	106.11	90.52 ± 0.47	83.52 ± 0.74	78.05 ± 1.27	65.32 ± 1.55
attUNet^18^	34.88	50.97	90.40 ± 0.40	83.22 ± 0.63	76.47 ± 4.34	62.73 ± 5.36
UCTransNet^9^	66.22	32.87	90.19 ± 0.43	82.91 ± 0.63	77.23 ± 0.59	64.09 ± 0.62
MSCA-Net^35^	27.09	9.03	85.98 ± 0.44	76.67 ± 0.58	75.45 ± 0.49	60.83 ± 0.64
Ours	15.77	26.35	90.56 ± 0.54	83.47 ± 0.81	79.07 ± 1.01	65.98 ± 1.06

**Figure 3:**
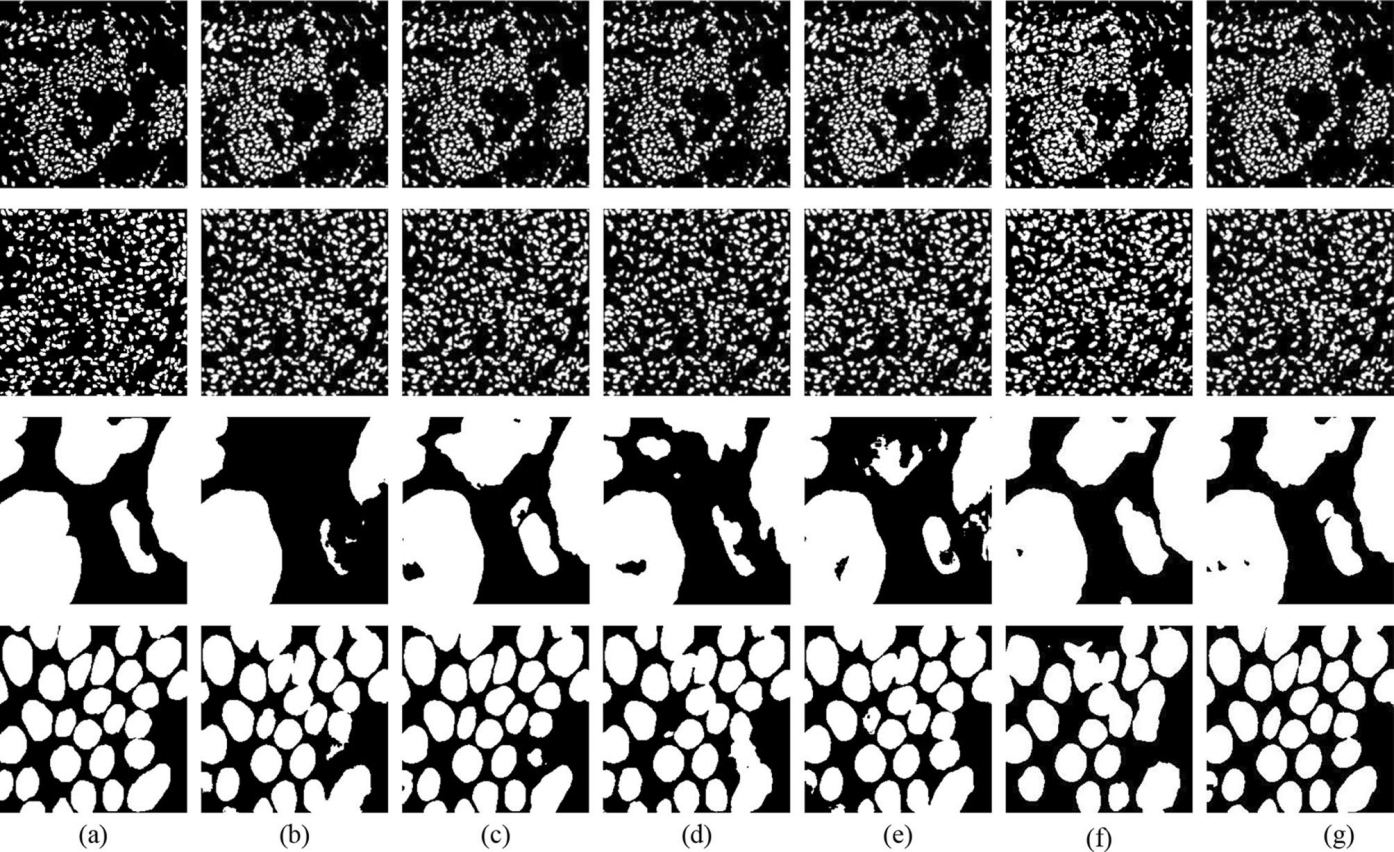
Illustration of the proposed TBSFF.

Figure 3 shows the visualization of segmentation results of different models. It can be seen that TBSFF-UNet achieves excellent performance and is more accurate than other models. The salient areas of the boundary are also very coherent, which once again verifies the effectiveness and advancement of the TBSFF-UNet method.

### Ablation studies

The ablation studies on the proposed module are shown in Table 2. “Base+TBSFF” outperforms other networks on both the GlaS and MoNuSeg datasets, which also proves the accuracy of our proposed module. Also, it shows that contextual information fusion is necessary to improve segmentation performance.

The previous experiments prove that adding skip connection and TBSFF module effectively improves network performance. To further evaluate the improvement, we spliced skip connections and adjusted the number of channels through convolution to exclude the impact of TBSFF on network performance. The model is defined as UNet+CAT. As can be seen from the experimental results in Table 2, the UNet+CAT model performs better than the UNet network on both GlaS and MoNuSeg datasets, which verifies the necessity of skip connections to improve network performance and also proves that the skip connections we added It is effective.

**Table 2:** Ablation experiments on GlaS and MoNuSeg datasets.

Method	Param (M)	FlOPs (G)	GlaS		MoNuSeg	
			Dice (%)	IoU (%)	Dice (%)	IoU (%)
Baseline (UNet)	13.40	23.74	89.42 ± 0.80	81.77 ± 1.31	77.53 ± 0.45	64.38 ± 0.53
Baseline+CAT	15.96	26.82	89.94 ± 0.29	82.64 ± 0.51	77.55 ± 1.29	64.41 ± 1.51
Baseline+TBSFF	15.77	26.35	90.56 ± 0.54	83.47 ± 0.81	79.07 ± 1.01	65.98 ± 1.06

To check the TBSFF module, we define UNet with the TBSFF module as Baseline+TBSFF and compare it with the Baseline and Baseline+CAT models. As can be seen from Table 2, on the GlaS and MoNuSeg data sets, the performance of Baseline+TBSFF is significantly better than Baseline and Baseline+CAT, which means that the TBSFF module has a positive effect on improving the network.

## Discussion

Medical image segmentation plays a vital role in the field of digital pathology. This paper proposes a contextual information fusion-based TBSFF-UNet network to provide a stable and reliable medical image segmentation method. It is validated on the GlaS and MoNuSeg benchmark datasets, and achieves a 90.56 dice coefficient and 83.47IoU on the dataset and 79.07dice and 65.98IoU on the MoNuSeg dataset. The network designed in this paper successfully extracts more effective information from the feature map by the encoder and narrows the semantic gap of contextual information. The advantages of the proposed model are demonstrated through rich experiments and in-depth analysis.

However, the model in this paper still has some shortcomings. For example, when the samples are seriously unbalanced, the algorithm’s performance is general. In the future, we will solve this problem by integrating Transformer and GAN networks into the network.

## Data Availability

The datasets generated or analyzed during this study are available at the MoNuSeg challenge repository, GlaS dataset repository (https://monuseg.grand-challenge.org/Data/, https://paperswithcode.com/dataset/glas).
